# Critical value in surgical pathology: evaluating the current status in a multicenter study

**DOI:** 10.1186/s13000-023-01342-8

**Published:** 2023-04-27

**Authors:** Neda Soleimani, Atefe Zare Sheibani, Samira Khajeh, Sahand Mohammadzadeh, Negar Taheri, Maral Mokhtari, Mahsa Farhadi, Zahra Hajizade, Saeideh Khaleghpanah, Sima Dehghani

**Affiliations:** 1 Department of Pathology, Shiraz Medical School, Shiraz University of Medical Sciences, Shiraz, Iran; 2 Department of Pathology, Shiraz Transplant Center, Abu Ali Sina Hospital, Shiraz University of Medical Sciences, Shiraz, Iran

**Keywords:** Critical value, Critical, Unexpected, Surgical pathology

## Abstract

**Background:**

The concept of critical value is not evident in surgical pathology, and there is no established protocol for determining, reporting, and documenting these results.

**Materials and methods:**

A questionnaire was designed regarding critical value in surgical pathology, and all pathologists and some clinicians from five laboratories were asked to participate through an invitation link. The most important items were selected, and all pathologists were instructed to follow a standard operating procedure to deal with critical results for a year.

**Results:**

A total of 43 pathologists and 44 non-pathologists participated in the study. Some critical or unexpected items were selected. Most participants agreed that the optimal time to announce critical reports is within 24 h of establishing the final diagnosis, and a phone call was the most dependable communication option. In addition, the most qualified recipients were the attending physicians. Therefore, a written policy was implemented for a year. One hundred seventy-seven critical or unexpected cases (0.5%) were detected. Mucormycosis and *cytomegalovirus (CMV)* were the most frequent critical cases.

**Conclusion:**

There are no set criteria for critical items or the reporting process in surgical pathology. It is possible to establish more uniform norms for reporting these cases by boosting pertinent research efforts and recruiting more pathologists and physicians. Additionally, it is advised that each medical facility compile its own unique critical or unexpected diagnosis list.

## Introduction

In 1972, Lundberg first proposed the concept of critical value [1]. A critical value refers to a laboratory finding outside the normal range that might constitute an immediate health risk that would be otherwise difficult to detect [2]. It is also known as "critical diagnosis," "urgent diagnosis," and treatable, immediately life-threatening diagnosis." Regardless of the attributed terms, an immediate report to a healthcare provider is necessary for taking the required medical actions [3].

In surgical pathology, turnaround time is variable from 2 to 14 days, involving tissue processing, slide preparation, microscopic evaluation, and the typing and signing of reports. In these reports, there are some critical results that demand rapid reporting for rapid intervention before routine reporting. Therefore, clear cutoff points must be developed to differentiate between life-threatening conditions and those that can be managed in routine practice [4, 5]. In addition to critical diagnoses, there are a few diagnoses in surgical pathology that are unusual or unexpected and should be addressed during treatment, although not as immediately as the critical ones. These results are referred to as "significant, unexpected diagnoses" [6].

The concept of critical value is quite evident when dealing with numerical data in clinical pathology; nevertheless, surgical pathology is information-sensitive, and surgical pathologists are involved in the interpretation of findings rather than numerical data [5, 7]. Non-pathologists and pathologists have quite different expectations, and these differing expectations might result in miscommunication and patient harm as a result [8]. Moreover, there are no clear guidelines, and research on this topic is limited. In the absence of such guidelines, the surgical pathologist's expertise and judgment determine when immediate physician contact is warranted. Furthermore, despite the critical value in clinical pathology, which is completely well-known, respected, and documented, many laboratories do not have proper estimation and documentation plans for critical values in surgical pathology [5, 9].

With this background in mind, the present study aimed to reach an agreement regarding the determination, documentation, and reporting of critical or unexpected surgical pathology results in centers affiliated with Shiraz University of Medical Sciences (SUMS), Shiraz, Iran, to calculate the annual frequency of these findings and evaluate the necessity of policy implementation.

## Materials and methods

This study was conducted in five surgical laboratories of the SUMS Pathology Department, which conduct pathological assessments in various medical fields. Centers 1 and 2 served as general centers; nevertheless, Centers 3, 4, and 5 were specialist centers for otorhinolaryngology, gynecology (GYN), and transplantation. This study was carried out according to the tenets of the Declaration of Helsinki after obtaining approval from the Ethics Committee of SUMS (IR.SUMS.MED.REC.1400.081). As there were no established criteria for critical or unexpected results in the studied centers, a multiple-choice questionnaire was developed. The list of diagnoses was chosen by the authors according to previous surveys and the authors' experience to represent diagnoses that might be critical [3, 6–10] (Table 1).

**Table 1:** The multiple-choice questionnaire for the participants

Position, level of education, and specialty	
**Questions**	Answers
*Which of the following items should be regarded as a critical/unexpected result?	-No specific entities, wholly at the pathologists' choice -Malignancies associated with superior vena cava syndrome -Any infectious organism in an immunocompromised patient (CMV, herpes, fungus, etc.) -Vasculitis -large vessel in core biopsy specimens -Crescent in a kidney biopsy specimen -Severe rejection in transplant -Necrosis in transplant -All positive acid-fast bacilli results -Any infectious organisms in a lung biopsy -Bacteria in a heart valve or evidence of endocarditis -Fat in endometrial biopsy -Fat in the gastrointestinal biopsy -Herpes in a PAP test of a pregnant patient -Uterine content without villi or trophoblast -Mesothelial cells in the heart biopsy -Fecal matter in peritoneal fluid cytology -Significant discrepancy between the intraoperative frozen diagnosis and the final diagnosis -Significant discrepancy between on-site cytology interpretation and final diagnosis -Significant discrepancy in consultation material between the original and consultation diagnosis -Significant specimen handling/processing issue (e.g., specimen lost or destroyed) -Amended and addendum reports with significant clinical impact
What is the optimal time to announce a critical/unexpected result?	-Within an hour of the final diagnosis interpretation -Within 24 h of the final diagnosis interpretation -There is no set time expectation
Which option is the most trustworthy way to communicate about a critical/unexpected result?	-In-person meeting -Phone call -Text message -E-mail
Who should be the most acceptable recipient to receive a critical/unexpected result?	-Attending physicians -Residents/fellows -Nurses -Office/administrative staff
What is the recommended approach for documentation?	-All cases should be documented, along with the date and time of reporting and the recipient's name -It is not necessary to document any report
What is the acceptable concept for a significant unexpected diagnosis?	-A diagnosis that is inconsistent with the clinical data -A malignant diagnosis without a previous history of malignancy -Diagnosis of a rare tumor -Diagnosis of a common tumor in an unusual location
What is the preferred plan for critical diagnoses that are not included in the checklist?	-Follow the same protocol as a critical- unexpected diagnosis -The individual pathologist should decide whether or not special communication with the clinical team is necessary -It should be communicated as other routine cases

^*^The ability to choose more than one item

All pathologists and some clinicians with various subspecialties in the five centers were asked to participate in this study using an invitation link. The most well-liked items for each question were selected to establish a standard operating procedure (SOP) for the determination, documentation, and reporting of critical or unexpected pathology results. Afterward, all pathologists in the study center were asked to follow this SOP. The statistics were compiled from all centers at the end of the year. Microsoft Excel (version 2016) was used to analyze the recorded data.

## Results

**Fig. 1:**
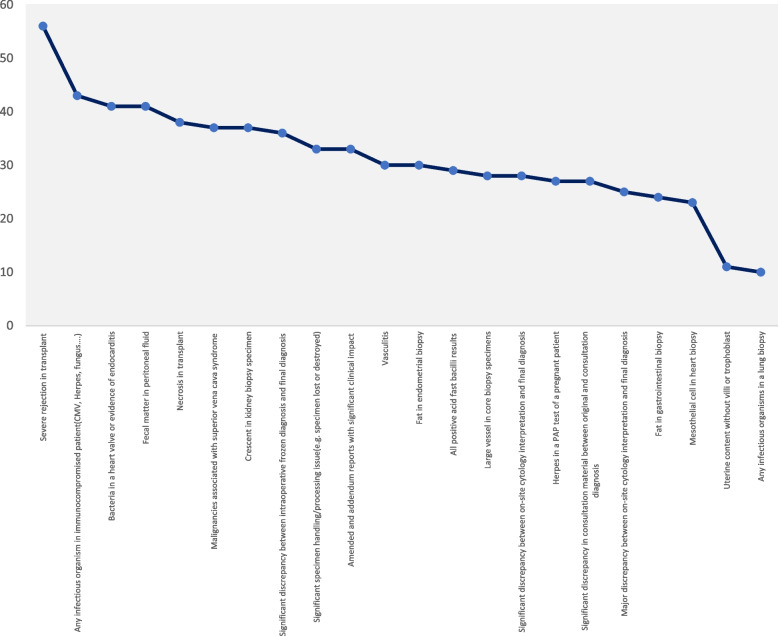
The participants' responses to the first question

Among 340 physicians (60 pathologists and 280 non-pathologists), a total of 87 physicians, including 43 pathologists and 44 non-pathologists, with subspecialties in general surgery (*n* = 15), GYN (*n* = 6), dermatology (*n* = 4), otorhinolaryngology (*n* = 3), urology (*n* = 2), neurosurgery (*n* = 2), internal medicine (*n* = 6), pediatric medicine (*n* = 3), and general medicine (*n* = 3) participated in this investigation. Overall, 37%, 5%, 33%, and 5% of the participants were residents, fellows, non-attending specialists, and general practitioners, respectively. Nearly 20% of the participants were attending physicians. The acceptable critical items are ranked from the most popular to the least popular in Fig. 1.

Most participants (64%) agreed that the optimal time to announce critical or unexpected reports is within 24 h of establishing the final diagnosis. In addition, phone calls (36%) and in-person meetings (31% each) were the most dependable communication options. The most qualified recipients of critical or unexpected results were the attending physicians, followed by residents and fellows. Moreover, most participants (94%) believed all critical or unexpected cases needed to be documented.

The concept of an unexpected diagnosis was defined as an inconsistent finding with the clinical information. Finally, most participants believed that in unpredictable situations, the individual pathologist should decide whether certain communication with the clinical team is necessary. Based on their responses, a written policy was selected and implemented for one year while documented data were collected and analyzed.

Among 33,934 pathology reports from five hospitals, 177 critical or unexpected cases (0.5%) were detected (Table 2), all of which were contacted and documented. Table 3 shows the number of critical or unexpected cases stratified by each center.

**Table 2:** Details of documented critical/unexpected cases

Critical results			
**Diagnosis**	**Total**	**Special cases**	**Numbers**
Mucormycosis infection	126	Sinonasal cavity	107
		Skin	5
		Orbit	6
		Small bowel	2
		Colon	1
		Stomach	1
		External ear	1
		Lung	1
		Bone	1
		Omentum	1
CMV infection	13	Colon	7
		Liver	2
		Esophagus	1
		Duodenum	1
		Stomach	1
		Lymph node	1
Other fungal infections	9	Brain	3
		Orbit	2
		Colon	2
		transplanted liver	1
		transplanted kidney	1
Severe rejection of transplanted organs	8	Liver	5
		Heart	2
		Kidney	1
Polyoma BKV infection Necrosis in transplanted organs	4	Transplanted kidney	4
Fat in endometrial biopsies	1	Liver	1
Fat in endometrial biopsies	1	Endometrium	1
SVC syndrome caused by malignancy	1	Mediastinal lymphoma	1
**Unexpected results**			
**Diagnosis**	**Total**	**Special cases**	**Numbers**
The clinically significant change in a frozen section diagnosis after reviewing permanent sections	7	Benign to malignant diagnosis	2
		Involvement of SLN in breast cancer in permanent sections	5
Pathologic findings that are significantly discordant with the preoperative diagnosis	7	A benign diagnosis with a preoperative diagnosis of malignancy	1
		Malignant diagnosis with the preoperative diagnosis of a benign lesion	6

*BKV* BK virus *SVC* Superior vena cava, *SLN* Sentinel lymph node

**Table 3:** Critical/unexpected cases stratified by each center

Centers	Subspecialty	Total pathology cases	Total critical / Unexpected results (%)	**The most common critical/unexpected case**
**1**	General	8978	57(0.6)	Mucormycosis
**2**	General	10,000	18(0.2)	The clinically significant change in a frozen section diagnosis after reviewing permanent sections
**3**	Otorhinolaryngology	2514	76(3)	Mucormycosis
**4**	Gynecology	4439	1(0.02)	Fat in endometrial biopsies
**5**	Transplantation	8003	24(0.3)	Severe rejection of transplanted organs
**Total Cases**		**33,934**	**177(0.5)**	Mucormycosis

## Discussion

One of the most critical functions of pathology reports or laboratory services is to facilitate clear, accurate, and rapid communication of critical test results (critical values) with care providers. Critical diagnoses refer to those that might have an immediate impact on patient care. An example of a critical diagnosis is finding a serious infection (e.g., CMV) in an immunocompromised individual. Significant, unexpected diagnoses should be both significant and unexpected, relying heavily on the pathologist's experience and judgment for identification. An example of a significant, unexpected diagnosis is finding a carcinoma in a uterus removed for leiomyoma [10–12].

According to Rosai and Ackerman's Surgical Pathology book, "when an urgent decision needs to be made based on pathological findings, the clinician should not wait for the information to reach him/her in a routinely typewritten report!" [10, 13]. In the literature, the number of studies evaluating the necessity of determining and reporting critical or unexpected pathology results is quite limited. In the annual meeting of the Iranian Society of Pathology in Tehran, Iran, Mireskadari reported on a study of 147 pathologists to determine which findings should be considered critical in surgical pathology. Nearly 90 different conditions were extracted from the aforementioned survey [5].

In 2004, Pereira et al. conducted a retrospective review and survey of 2,659 surgical pathology reports based on the perceptions of five clinicians and 11 pathologists regarding critical values in surgical pathology. They identified 13 critical cases (0.49%). Moreover, 4 out of these 13 reports documented phone calls to clinicians (in most cases, at least one day before the final sign-out) [10].

Pathologists should reach an agreement with their clinician colleagues on what kinds of diagnoses are regarded as critical. Moreover, effective communication and proper documentation in pathology reports are the key components of establishing a critical diagnosis policy. The consequences of a delay or failure in communicating critical diagnoses might be devastating. The main reason for establishing a policy for critical or unexpected diagnoses in surgical pathology is to ensure that the written report is not overlooked. Verbal communication would hasten the reporting process. However, since the communication between pathologists and clinicians is frequently suboptimal and might result in misunderstandings, all reports should be conveyed in writing. Furthermore, semi-automated reporting via special codes can improve the quality of patient care [2, 7, 14].

According to the Association of Directors of Anatomic and Surgical Pathology (ADASP), the establishment of critical diagnosis guidelines for anatomic pathology represents practice improvement and patient safety initiatives. The ADASP also recognized that a generic critical diagnosis guideline in anatomic pathology should only be used as a template because the list needs to be customized in each laboratory following consultation with relevant clinical service providers. In the meantime, the College of American Pathologists (CAP) added checklist items GEN.41320 and GEN.41330 to its Laboratory General Checklist, necessitating laboratories to have written procedures for immediate physician notifications when the results fall outside specific critical ranges and to document notification alerts for these results [11, 15, 16].

The Joint Commission on Accreditation of Healthcare Organizations and the CAP surveyed 1,130 laboratories to determine the current policies and practices for critical diagnoses in anatomic pathology. The survey results demonstrated that 75% of laboratories had a written policy for critical diagnoses in anatomic pathology; nevertheless, only 30% had a list of specific examples of critical diagnoses. Additionally, the effective communication and documentation of critical diagnoses in anatomic pathology are not well addressed in the literature [17]. A study by Coffin in 2007 showed that 9.4% of pediatric surgical pathology accessions were critical and that nearly 80% had been reported and documented before policy implementation. Based on the findings of the aforementioned study, after policy implementation, 97.3% (402/413) of the cases were verbally reported and documented [6].

With individual center rates ranging from 0.02% to 3%, on average, 0.5% of the cases in this study after policy implementation were critical or unexpected, comparable to Pereira's survey results.

Opportunistic infections caused by Mucormycosis and CMV were the most frequent critical or unexpected cases in the centers investigated for this study. However, there might be some variations in infectious organisms between centers with different geographic and health conditions.

Although the otorhinolaryngology center and one general center reported the majority of discovered cases, respectively, other centers also reported significant critical cases, such as severe rejections and necrosis of transplanted organs in the transplantation center. Additionally, the most prevalent critical or unexpected cases differed among centers, highlighting the importance of list formation and policy implementation for every center separately.

The fact that this recently implemented policy for determining, documenting, and reporting critical or unexpected pathology results was developed based on input from pathologists and non-pathologists (clinicians) was advantageous; however, it is still impossible to guarantee that all critical or unexpected results will be addressed. Generally, developing a dynamic list and policy emphasizing both pathologists' and physicians' views, in addition to an ongoing evaluation of success, can be an ideal approach for dealing with critical or unexpected cases.

## Conclusion

Although the concept of critical value in surgical pathology has been recently accepted by most laboratories, there is no standardization for critical items. It might be possible to develop more uniform norms for the determination, reporting, and documentation of these cases by expanding relevant research and recruiting more pathologists and physicians. Additionally, each medical facility is recommended to compile its own unique critical or unexpected diagnosis list and SOP to deal with surgical pathology findings, as these cases vary among medical facilities.

## Data Availability

All data generated or analysed during this study are included in this published article.

## Abbreviations

TILT: Treatable immediately, Life-Threatening
TAT: Turnaround time
SUMS: Shiraz University of Medical Sciences
GYN: Gynecology
SOP: Standard operating procedure
CMV: Cytomegalovirus
BKV: BK virus
SLN: Sentinel lymph node
SVC: Superior vena cava
ADASP: Association of Directors of Anatomic and Surgical Pathology
CAP: College of American pathologists
